# 

**Published:** 1889-07

**Authors:** 


### CHANGE OF ADDRESS.

ONWARD and upward has ever been the watchword of the publishers of the JOURNAL, who have spared neither pains nor money in the past to make it the best dental journal published in America. The present number begins a second year under our management, and we do not think that any one can say that we have not made each succeeding issue better than its predecessor,—a step in advance. There has been nothing to deprecate in the quantity or quality of the matter furnished during the past year, but we have not always been satisfied with the typography and press-work. We have now made a change in printers, and hereafter the JOURNAL will be issued by the well-known and thoroughly reliable house, The J. B. Lippincott Company, 716 Filbert Street. We feel that there can now be no fault found with the JOURNAL in any respect, and that the matter furnished will be presented in the best dress that is possible for any journal to obtain. The old and familiar pen, scroll, retort, skull, and name—"Independent Practitioner"—will be discarded, and a plain but bold face presented for your approval. The INTERNATIONAL will be just as independent as when it bore its former title.

The full-fledged JOURNAL is now before you, having cast off the last vestige of its previous habiliment. You have witnessed its evolution during the year past, and now behold the spirit of our dreams. What is your verdict? Is it worth another year's subscription? for that of some of our readers expired with the June number. We have sent you the July number in order that you might see the JOURNAL in its new dress before we cross your name from off our subscription-list, which we shall surely have to do, if we do not hear from you before the August number is out. Send in your subscription for another year, and invite one of your neighbors to join you. You can send five dollars just as easy as two dollars and fifty cents. Please note the change of address, 716 Filbert Street.

